# Endoscopic transvaginal drainage and necrosectomy of presacral walled-off pancreatic necrosis

**DOI:** 10.1055/a-2127-4516

**Published:** 2023-08-01

**Authors:** Mia Prindahl Ærenlund, Lars Lindgaard, Srdan Novovic, Morten Laksáfoss Lauritsen, John Gásdal Karstensen, Palle Nordblad Schmidt

**Affiliations:** 1 Pancreatis Centre East, Gastro Unit, Copenhagen University Hospital – Amager and Hvidovre, Copenhagen, Denmark; 2 Department of Clinical Medicine, University of Copenhagen, Copenhagen, Denmark

Transluminal drainage and necrosectomy has become the preferred choice of treatment for complicated walled-off pancreatic necrosis (WON). Transgastric, transduodenal, transrectal, and transcolonic approaches have been described 1 2 3 4 , but in some cases the location of the WON prevents access through the gastrointestinal tract. We here describe a case of transvaginal drainage and necrosectomy.

The patient was a 50-year-old woman with Crohn’s disease and previous proctocolectomy. She underwent an endoscopic retrograde cholangiopancreatography (ERCP) for common bile duct stones. The procedure was complicated by severe post-ERCP pancreatitis. After 36 days, the patient developed a large WON surrounding the right kidney, extending into the pelvis and the right thigh ( Fig. 1 ).

**Fig. 1:**
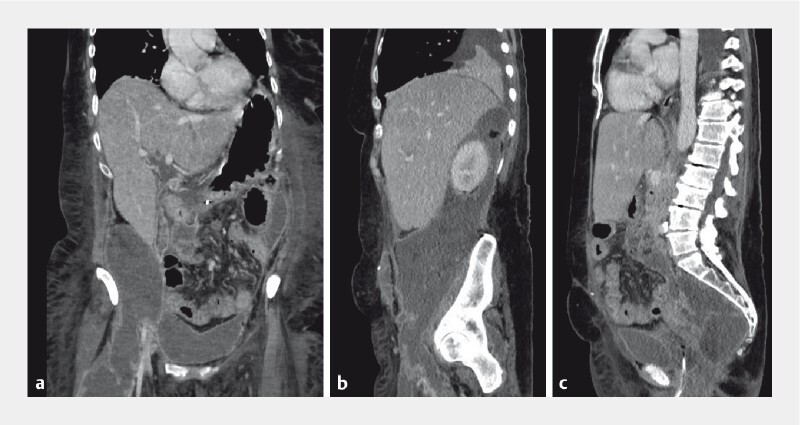
Contrast-enhanced computed tomography image of the abdomen 36 days after the onset of pancreatitis showing the distribution of necrosis in the: **a** coronal plane, with extension of the necrosis evident from the retroperitoneum into the right thigh; **b** sagittal plane, with perirenal and intrapelvic extension visible; **c** sagittal plane, with the presacral necrosis that was not accessible for video-assisted retroperitoneal debridement or other endoscopic transluminal approaches.

The WON was initially drained percutaneously as it was inaccessible from the gastrointestinal tract. After six video-assisted retroperitoneal debridement (VARD) procedures and continuous drainage via multiple percutaneous drains, the right-sided WON had resolved. A residual area of infected presacral necrosis was inaccessible for VARD; however, the patient developed a spontaneous fistula between this area of necrosis and the vagina. Vaginoscopy was performed using a therapeutic gastroscope ( Video 1 ). Under fluoroscopic guidance, a contrast catheter with guidewire (VisiGlide2; Olympus, Hamburg, Germany) was introduced through the fistula into the area of necrosis. The fistula was balloon dilated to 10 mm and two 12-cm 7-Fr double-pigtail stents were inserted, along with a 7-Fr irrigation catheter ( Fig. 2 ; Video 1 ).

**Video 1** Endoscopic transvaginal drainage and necrosectomy is performed for an area of presacral walled-off pancreatic necrosis that was inaccessible from the gastrointestinal tract.


**Fig. 2:**
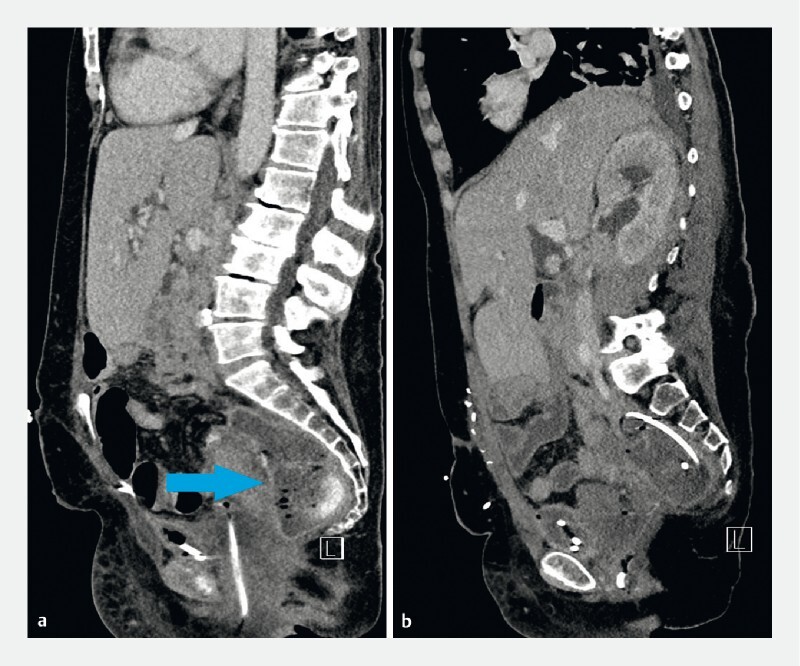
Contrast-enhanced computed tomography image of the abdomen showing: **a** on day 63 after the onset of pancreatitis (before the initial vaginoscopy), the area of presacral necrosis (arrow); **b** on day 75, the appearance 4 days after the initial vaginoscopy and drain insertion.

During three additional procedures, the vaginal fistula was gradually dilated up to 18.5 mm and extensive endoscopic necrosectomy was performed using polypectomy snares until the WON was free of debris. During the final procedure, two double-pigtail stents were inserted, which were removed 1 month later. The patient was discharged 81 days after the initial VARD procedure. Currently, 3 years after discharge, there has been no recurrence of the pancreatitis or pancreatic fluid collection and the patient has no vaginal complaints.

Endoscopy_UCTN_Code_TTT_1AT_2AF

## Notes

Endoscopy E-Videos

https://eref.thieme.de/e-videos
*E-Videos* is an open access online section of the journal *Endoscopy* , reporting on interesting cases and new techniques in gastroenterological endoscopy. All papers include a high-quality video and are published with a Creative Commons CC-BY license. Endoscopy E-Videos qualify for HINARI discounts and waivers and eligibility is automatically checked during the submission process. We grant 100% waivers to articles whose corresponding authors are based in Group A countries and 50% waivers to those who are based in Group B countries as classified by Research4Life (see: https://www.research4life.org/access/eligibility/ ). This section has its own submission website at https://mc.manuscriptcentral.com/e-videos
